# Influences of applying Yunnan Baiyao aerosol combined with finger rehabilitation exercises on local complications of radial artery after transradial coronary intervention

**DOI:** 10.3389/fcvm.2026.1791991

**Published:** 2026-07-27

**Authors:** Bo Pan, Hechun Shen, Jielin Yuan, Hongxin Ou, Jianrong Huang

**Affiliations:** 1 Department of Cardiovascular Internal Medicine Ward One, The Second Nanning People's Hospital, Nanning, Guangxi, China; 2 Department of Neurosurgery, The Second Nanning People's Hospital, Nanning, Guangxi, China

**Keywords:** finger rehabilitation exercises, pain, radial artery occlusion, swelling, transradial coronary intervention, Yunnan Baiyao aerosol

## Abstract

**Aim:**

Transradial coronary intervention, while advantageous, is frequently associated with local complications such as pain, swelling, and radial artery occlusion (RAO). Conventional management methods, including limb elevation and compression, often have limitations in efficacy and patient comfort. This study investigated the effectiveness of a combined intervention using Yunnan Baiyao aerosol and structured finger rehabilitation exercises on these local complications.

**Methods:**

From April 2023 to April 2025, 100 patients undergoing transradial coronary intervention were enrolled and randomly assigned to either an intervention group or a control group. The control group received standard care with enhanced limb elevation and compression bandaging. The intervention group received, in addition to standard care, a protocol involving Yunnan Baiyao aerosol application and nurse-administered finger rehabilitation exercises. Outcomes, including pain (Numerical Rating Scale), hand swelling (palm circumference), postoperative bleeding, RAO (via ultrasound at 72 h), comfort level (General Comfort Questionnaire), and quality of life (SF-36), were assessed at predefined time points.

**Results:**

The intervention group presented significantly lower pain scores and smaller palm circumference on postoperative days 1, 2, and 3 compared to the control group (all *P* < 0.01). The incidence of RAO at 72 h was markedly lower in the intervention group (2.00% vs. 18.00%, *P* < 0.05). The intervention group reported significantly higher scores for comfort and quality of life on day 3 (*P* < 0.05). No significant difference was reported in postoperative bleeding rates between the groups (*P* > 0.05).

**Conclusion:**

The combined application of Yunnan Baiyao aerosol and finger rehabilitation exercises is an effective and safe nursing intervention. It significantly alleviates post-procedural pain and swelling, decreases the early incidence of radial artery occlusion, as well as improves patient comfort and short-term subjective well-being (assessed at day 3) following transradial coronary intervention, without increasing bleeding risk. These findings, however, are limited to the acute recovery phase; long-term quality of life outcomes were not evaluated.

## Introduction

Transradial coronary intervention has emerged as an extensively adopted, advanced approach for the diagnosis and treatment of coronary heart disease, recognized for its advantages of minimal trauma, lower complication rates, and enhanced patient comfort due to unrestricted positioning post-procedure (1). Despite these benefits, local complications such as limb pain and swelling remain prevalent after radial artery access. Radial artery occlusion (RAO) is one of the most common complications following trans-radial procedures. A recent 2024 systematic review and meta-analysis of 41 studies involving 30,020 patients reported an overall RAO incidence of 13% (95% CI: 9%–16%), with early RAO (within 24 h) incidence of 14% (95% CI: 10%–18%) (2). RAO primarily results from prolonged hemostatic compression or insufficient hemostasis leading to bleeding and related sequelae (3). Anatomically distant from the heart, the hand exhibits comparatively slower local blood circulation and cellular turnover, which predisposes to fluid accumulation in extracellular and interstitial spaces, exacerbating localized swelling (4). Persistent fluid retention can further impair lymphatic drainage, and when compounded by postoperative inflammatory responses, may lead to venous and lymphatic stasis, compromised microcirculation, and in severe cases, localized ischemia or necrosis (5).

Conventional management strategies, including limb elevation, compression bandaging, and topical agents such as magnesium sulfate wet compresses, offer limited efficacy due to delayed onset of action, practical constraints in maintaining continuous application, and potential skin irritation (6). While patent hemostasis techniques have been recommended to reduce RAO rates, the optimal hemostasis protocol remains debated, and many centers still rely on conventional compression methods (7). A recent 2025 network meta-analysis of randomized controlled trials on radial hemostasis devices confirmed that the incidence of RAO following hemostasis varies widely (1%–33%), and no single device has demonstrated clear superiority (8).

In contrast, traditional Chinese medicine attributes swelling to impaired fluid metabolism and often employs physical interventions such as finger exercises and acupoint stimulation to promote circulation, relieve pain, and reduce edema (9). A 2023 randomized clinical trial comparing three hand exercise regimens (finger exercise, acupoint massage, and handgrip exercise) in 102 patients after transradial cardiac catheterization found that all three interventions significantly reduced limb edema and pain, with handgrip exercise showing superior efficacy in edema reduction (10). These findings support the potential value of structured finger rehabilitation exercises in post-procedural care.

Yunnan Baiyao, a renowned herbal formulation, has been reported in the literature to possess hemostatic, anti-thrombotic, circulation-promoting, and anti-inflammatory properties (11). A 2025 comprehensive review systematically summarized the pharmacological mechanisms of Yunnan Baiyao, including platelet activation, arachidonic acid metabolism regulation, and autophagy regulation, through which it exerts hemostatic, anti-inflammatory, analgesic, and wound-healing effects (12). Its modern aerosolized form provides sustained topical delivery, enhancing its potential for managing trauma-related swelling and pain. A 2014 clinical study demonstrated that Yunnan Baiyao aerosol effectively relieved pain and swelling in patients with subcutaneous hematoma after percutaneous coronary intervention, with a total effective rate of 97.1% (13). Preliminary studies suggest that Yunnan Baiyao aerosol may improve microcirculation and modulate platelet function to alleviate edema (14).

Building on these principles, this study aims to assess the combined effect of Yunnan Baiyao aerosol application and structured finger rehabilitation exercises on alleviating local complications—specifically pain, swelling, and RAO—following transradial coronary intervention.

## Methods

### Study design

From April 2023 to April 2025, one hundred patients who underwent transradial coronary intervention in the cardiovascular department of our hospital were included as the research subjects. This study was approved by the hospital's ethics committee, and the registration number from the National Medical Research Registration and Filing Information System was MR-45-26-036154. All patients and their families signed the informed consent form.

Inclusion criteria: (1) Preoperative Allen Test was positive, with good coagulation function, and postoperative condition was stable without severe functional impairments of important organs such as liver, brain, and kidney; (2) No postoperative cognitive impairment or unclear speech expression; (3) Pain and swelling occurred in the punctured limb after surgery, and the skin was without severe infection or ulceration; (4) The patient had good compliance.

Exclusion criteria: (1) Severe dysfunction of important organs such as the heart, liver, and kidneys; (2) Severe arrhythmia, electrolyte imbalance, abnormal coagulation function; (3) Mental abnormalities; (4) Allergy to Yunnan Baiyao; (5) Post-cerebral infarction limb movement dysfunction, forearm deformity, obvious swelling of the punctured limb before the operation, trauma; (6) Preoperative skin infection or ulceration.

### Sample size calculation

The sample size was calculated based on the pain score obtained from previous studies (15). To achieve an 80% test power and an effect size of 0.5, a total of 90 participants were required. Considering a dropout rate of 10%, an additional 10 patients were needed; thus, the number of participants selected for this study was 100.

### Randomization and blinding

Patients were randomly divided into the intervention group or the control group through a computer-based stratified randomization method. This process was kept confidential from both the researchers and the participants. Each group consisted of 2 individuals, with an equal number in both groups. The randomization sequence was generated by an administrative staff member who was not involved in the recruitment process and was done using an opaque sealed envelope. Only when potential participants met the criteria for random assignment would a graduate research investigator open the envelope.

Due to the visible nature of the topical aerosol application and the active finger exercises, it was not possible to blind the patients or the nursing staff delivering the intervention. Importantly, outcome assessors were also not blinded to group allocation.

### Methods

The control group received enhanced limb elevation and local compression bandaging care. The affected limb was elevated to a range of 40–60 degrees. Hemostasis was achieved using a radial compression device (TR Band, Terumo). The compression device was maintained for 6 h after sheath removal, according to the standard post-interventional protocol of our institution during the study period (April 2023–April 2025). Six hours after the operation, the compression hemostasis device was removed and replaced with elastic bandaging for compression. No finger rehabilitation exercises or Yunnan Baiyao aerosol were applied in the control group.

Based on the treatment of the control group, the intervention group received nursing care using Yunnan Baiyao aerosol combined with finger rehabilitation exercises.

### Finger rehabilitation exercises

All exercises were performed by nursing staff at the patient's bedside. The protocol was divided into two phases based on the status of the radial compression hemostasis device.

#### Phase 1 (compression device in place, i.e., first 6 h post-PCI)

Exercises were passive or very gentle assisted active maneuvers only, designed to promote venous/lymphatic return without applying significant force to the puncture site.

Timing: Initiated 60 min after the patient returned to the ward.

Frequency: Every 30 min before each scheduled decompression of the hemostasis device (typically 2 decompressions before final removal), and every 60 min between decompressions. Each session lasted 3–5 min.

Maneuvers during Phase 1 (no active gripping or resistance):

1. Massaging the palm and back of the hand (gentle rubbing, 5 times).
2. Massaging each finger from base to tip (kneading, 5–10 s per finger).
3. Passive finger counting: The nurse gently extended and flexed the patient's fingers one by one (patient relaxed, no active effort).
4. Finger flicking (passive): The nurse pressed the patient's fingertip into an arch and released—the patient did not actively spring the finger.
5. Acupoint massage (Hegu, Houxi)—light pressure only, no vigorous kneading.

#### Phase 2 (after compression device removal, i.e., from 6 h post-PCI through day 3)

Timing: Exercises were performed four times daily (morning, noon, afternoon, and night) for a total of 3 days.

Maneuvers during Phase 2 (active exercises permitted):

1. Massaging palm and back of hand (as above).
2. Massaging five fingers (as above).
3. Active finger counting: The patient actively made a fist, then extended fingers one by one, then flexed back to a fist.
4. Ball grasping (gentle): The patient grasped a soft, low-resistance massage ball (diameter 5 cm, foam material) and performed a very gentle squeeze-release cycle (10 times). The nurse instructed the patient to avoid forceful gripping and to stop immediately if any increase in pain or oozing at the puncture site was noticed.
5. Active finger flicking: The patient pressed their own fingertip into an arch and released.
6. Acupoint massage (Hegu, Houxi)—the patient or nurse applied gentle pressure (20 s per point).

**Rationale for safety:** During Phase 1 (compression device in place), all exercises were either passive (nurse-moved) or involved only light touch without active patient effort, thus no increase in radial artery pressure or shear force at the puncture site. During Phase 2, the compression device had been removed, and hemostasis was already achieved (confirmed by absence of bleeding or hematoma). The active exercises were performed with low intensity, and the patient's pain and the puncture site were monitored before and after each session.

**Hospital stay and compliance monitoring:** All patients in this study remained hospitalized for at least 3 days after the PCI procedure, as per the standard post-interventional observation protocol of our institution. Therefore, all scheduled exercise sessions (four times daily for 3 days) were administered and directly supervised by the nursing staff at the bedside. For the few patients (*n* = 3 in the intervention group) who were discharged on day 2 or early day 3, they received an additional 1 h training session for family members, a printed instruction sheet with pictures, and a daily log to record exercise completion; these patients were followed up by telephone on day 3 to confirm adherence. Compliance was high (100% for in-hospital sessions, and the 3 discharged patients reported completing all prescribed sessions).

## Yunnan Baiyao aerosol

The nursing staff used a two-component Yunnan Baiyao aerosol system: (1) the protective liquid (for acute analgesia and anti-inflammation) and (2) the Yunnan Baiyao aerosol (for promoting circulation and resolving stasis). The application was initiated after removal of the compression hemostasis device (6 h after surgery) and repeated four times daily (morning, noon, afternoon, and night) for a 3-day treatment course.

## Application protocol

The sequential application (protective liquid first, followed by the aerosol after drying) follows the manufacturer's standard instructions and is the routine clinical practice for this product (Yunnan Baiyao Group Co., Ltd., product insert, 2020). The protective liquid consists predominantly of volatile solvents that evaporate rapidly, forming a non-occlusive layer that does not impede subsequent drug permeation.

Protective liquid: First, the nursing staff sprayed the protective liquid onto the local area of pain and swelling, with a spray range slightly larger than the affected area (approximately 5–10 cm beyond the visible margin of swelling). Each application consisted of 1–2 actuations (approximately 0.1–0.2 mL per actuation).

Drying period: The protective liquid was allowed to dry completely on the skin (approximately 30 s).

Yunnan Baiyao aerosol: After the protective liquid had dried, the nursing staff sprayed the Yunnan Baiyao aerosol onto the same area, using the same number of actuations (1, 2) and coverage range.

Precautions: The nursing staff avoided spraying onto broken skin or open wounds and closely monitored the patient for any signs of local allergic reactions (e.g., rash, itching, increased redness).

During the treatment period, the nursing staff closely monitored the radial artery pulse of the affected limb of the patient, as well as changes in the temperature, color, sensation, and opposition function of the palm.

## Observation indicators

1. The Numerical Rating Scale (NRS) method was adopted for pain assessment before the intervention and on the 1st, 2nd, and 3rd days after the intervention (16). A scale of 0–10 was used to determine the intensity of pain. A score of 0 represented no pain; 1–3 points represented mild pain; 4–6 points represented moderate pain; 7–9 points represented severe pain; and 10 points signified excruciating pain.
2. The swelling of the operated limb of the patient was represented by measuring the palm circumference before the intervention and on the 1st, 2nd, and 3rd days after the intervention. A tape measure of the same specification was used to measure the palm circumference (that is, with all fingers closed together, starting from the second phalanx of the thumb and measuring the horizontal length around the palm).
3. The degree of postoperative bleeding was determined according to the Christenson standard (17). The degree of bleeding was classified as follows: (a) No bleeding: no bleeding at the puncture site; (b) No obvious bleeding: the diameter of bleeding at the puncture site was ≤ 2 cm, and no hematoma was felt in the forearm or the diameter of the hematoma was ≤ 2 cm; (c) Obvious bleeding: the diameter of bleeding at the puncture site was >2 cm, and the diameter of the hematoma in the forearm was greater than 2 cm or re-pressurization for hemostasis was required. The trained hemostasis operation team members evaluated the degree of postoperative bleeding of the patients within 24 h after the surgery, and accurately recorded the results on the assessment form set up at the bedside.
4. The incidence of RAO 72 h after surgery was diagnosed by color Doppler ultrasound. All 100 enrolled patients underwent color Doppler ultrasound examination of the accessed radial artery at 72 h post-procedure. RAO was defined according to the standardized criteria established in consensus guidelines for transradial interventions (SCAI 2019 expert consensus): (a) complete RAO—absence of any antegrade flow signal in the radial artery on color Doppler ultrasound; (b) functional RAO—presence of only a punctate or speckled flow signal without continuous antegrade flow (previously described in the literature as “star-shaped” or “tiny speckled” flow). Both patterns were considered indicative of RAO, as punctate/speckled flow represents minimal residual flow that does not maintain clinically meaningful patency and has been consistently classified as occlusion in landmark trials such as the RAP and BEAT trials.
5. Before the intervention and on the 3rd days after the intervention, the General Comfort Questionnaire (GCQ) was used to assess their comfort level (18). The scale consisted of a total of 30 items, including 5 physiological items, 8 social and cultural items, 10 psychological items, and 7 environmental items. Each item was rated from 1 to 4 points, with a total score ranging from 0 to 120. The higher the score, the higher the level of comfort.
6. Before the intervention and on the 3rd days following the intervention, short-term subjective well-being during early recovery was evaluated using the Chinese version of the 36-Item Short Form Health Survey (SF-36) (19). While the SF-36 is typically used to assess quality of life over longer periods (weeks to months), in this study it was administered only at baseline and day 3 to capture patients' perception of their health status during the acute post-procedural phase. Therefore, the results reflect early recovery experiences rather than sustained quality of life. This scale consists of 8 dimensions: physical function, bodily pain, physical role, emotional role, mental health, vitality, social function, as well as overall health, with 36 items in total. The total score for each dimension ranges from 0 to 100, with higher scores indicating better quality of life.

## Statistical analysis

The statistical analysis of the data was carried out using SPSS version 23.0 software. For count data, the results were presented as the count of cases and corresponding percentages, with comparisons made utilizing either the *χ*² test. Measurement data that conformed to a normal distribution were expressed as the mean ± standard deviation (x¯ ± s), and group comparisons were conducted using the independent sample *t*-test. To assess intervention effects across various time points, repeated measures analysis of variance was employed. In instances where there was an observed interaction effect between the groups and time, separate analyses were performed to evaluate the individual effects of the groups and time. A statistically significant difference was defined as *P* < 0.05.

All time points (baseline, days 1, 2, and 3 for pain and swelling; baseline and day 3 for comfort and QoL) were pre-specified in the study protocol. For outcomes measured at multiple time points (pain and swelling), we used repeated measures ANOVA with post-hoc simple effects analysis to assess within-group changes from baseline. For outcomes measured at two time points (comfort and QoL), within-group changes were evaluated using paired *t*-tests, and between-group differences in change-from-baseline scores (post-intervention minus baseline) were analyzed using independent *t*-tests. Change-from-baseline data are presented as mean differences with 95% confidence intervals (CIs).

## Results

### General data

The mean age was 62.4 ± 8.3 years in the intervention group and 63.1 ± 7.9 years in the control group (*P* = 0.67). Male patients accounted for 58% (29/50) in the intervention group and 62% (31/50) in the control group (*P* = 0.68). Other baseline characteristics, including body mass index, comorbidities, and procedural details, were also comparable between groups (all *P* > 0.05). No significant differences were discovered in general data between the two groups (*P* > 0.05, Table 1).

**Table 1:** General data between the two groups.

Item	Control group (*n* = 50)	Intervention group (*n* = 50)	*χ*^2^/t	*P*
Gender			0.183	0.668
Male	33 (66.00)	35 (70.00)		
Female	17 (34.00)	15 (30.00)		
Age (years)	62.23 ± 7.25	62.30 ± 7.28	0.048	0.961
History of smoking			0.367	0.544
Yes	27 (54.00)	30 (60.00)		
No	23 (46.00)	20 (40.00)		
History of drinking			0.198	0.656
Yes	13 (26.00)	15 (30.00)		
No	37 (74.00)	35 (70.00)		
Hypertension			0.160	0.688
Yes	27 (54.00)	25 (50.00)		
No	23 (46.00)	25 (50.00)		
Diabetes			0.508	0.475
Yes	13 (26.00)	10 (20.00)		
No	37 (74.00)	40 (80.00)		

### Procedural characteristics

To assess potential confounding factors known to influence radial artery occlusion, we compared procedural details between the two groups. As shown in Table 2, there were no significant differences in the indication for PCI (stable angina, unstable angina, NSTEMI, STEMI), sheath size (5Fr vs. 6Fr), type and dose of intraprocedural anticoagulation (unfractionated heparin 70–100 U/kg vs. bivalirudin), or post-procedural antiplatelet therapy (aspirin + clopidogrel vs. aspirin + ticagrelor). All comparisons yielded *P* > 0.05, indicating that the two groups were well balanced with respect to these procedural variables.

**Table 2:** Comparison of procedural characteristics between the two groups.

Procedural characteristic	Control group (*n* = 50)	Intervention group (*n* = 50)	χ²/t	*P*
Indication for PCI			0.312	0.958
Stable angina	12 (24.0%)	11 (22.0%)		
Unstable angina	20 (40.0%)	22 (44.0%)		
NSTEMI	10 (20.0%)	9 (18.0%)		
STEMI	8 (16.0%)	8 (16.0%)		
Sheath size			0.178	0.673
5Fr	35 (70.0%)	33 (66.0%)		
6Fr	15 (30.0%)	17 (34.0%)		
Intraprocedural anticoagulation			0.367	0.544
Unfractionated heparin (70–100 U/kg)	42 (84.0%)	44 (88.0%)		
Bivalirudin	8 (16.0%)	6 (12.0%)		
Total heparin dose (U, mean ± SD)	5,680 ± 850	5,720 ± 820	0.242	0.809
Post-procedural antiplatelet therapy			0.090	0.764
Aspirin + clopidogrel	38 (76.0%)	39 (78.0%)		
Aspirin + ticagrelor	12 (24.0%)	11 (22.0%)		

### Degree of pain

**Figure 1:**
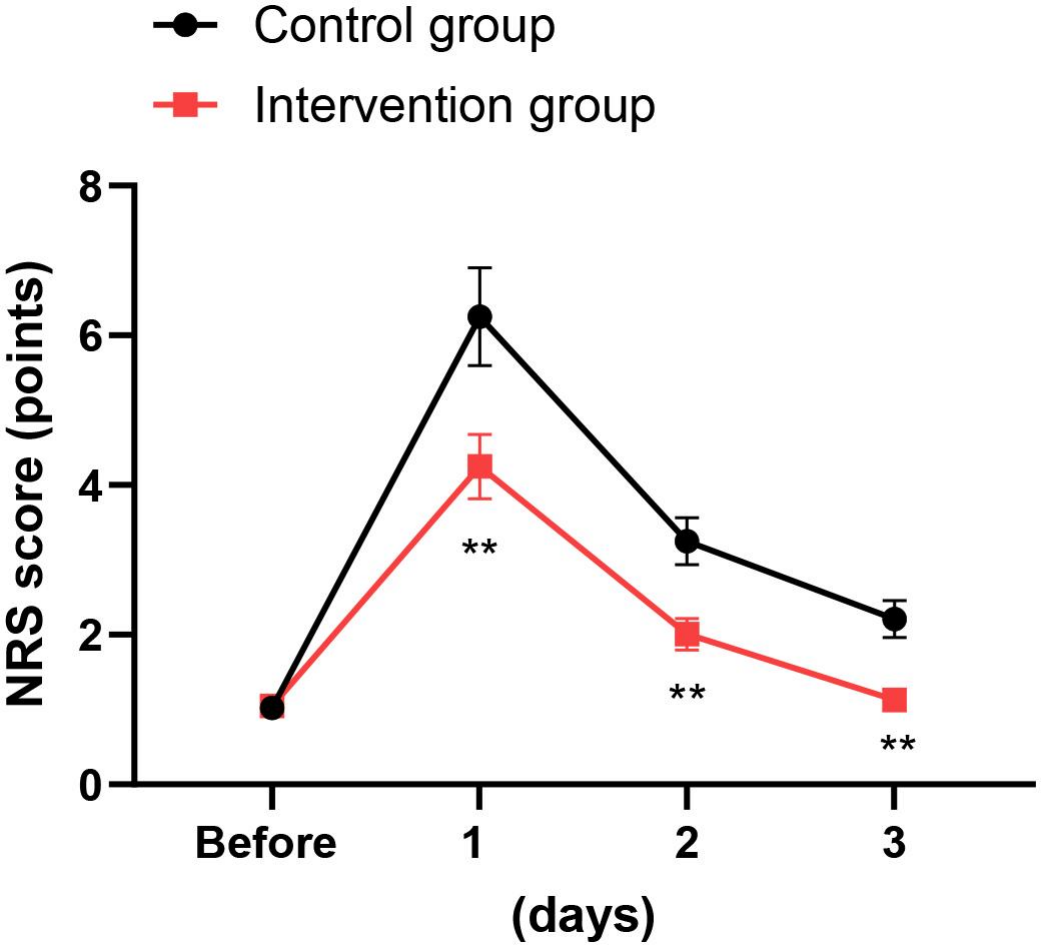
Degree of pain at different times between the two groups. **P < 0.01.

Prior to the intervention, no significant difference was reported in the NRS scores between the two groups (intervention: 5.8 ± 1.2, control: 5.7 ± 1.3, *P* = 0.68). On the 1st, 2nd, and 3rd days after the intervention, the NRS scores of the intervention group were lower than those of the control group (all *P* < 0.01, Figure 1). Specifically, on day 1: intervention 4.2 ± 1.1 vs. control 5.0 ± 1.2, mean difference −0.8, 95% CI: −1.2 to −0.4, *P* = 0.006; on day 2: intervention 2.9 ± 0.9 vs. control 4.1 ± 1.1, mean difference −1.2, 95% CI: −1.6 to −0.8, *P* < 0.001; on day 3: intervention 1.9 ± 0.8 vs. control 3.4 ± 1.1, mean difference −1.5, 95% CI: −1.9 to −1.1, *P* < 0.001.

Within-group analyses showed that both groups experienced significant reductions in NRS scores from baseline to day 3 (intervention group: mean change −3.9, 95% CI −4.3 to −3.5, *P* < 0.001; control group: mean change −2.3, 95% CI −2.7 to −1.9, *P* < 0.001). The reduction was significantly greater in the intervention group (between-group difference in change: −1.6, 95% CI −2.1 to −1.1, *P* < 0.01).

### Palm circumference

**Figure 2:**
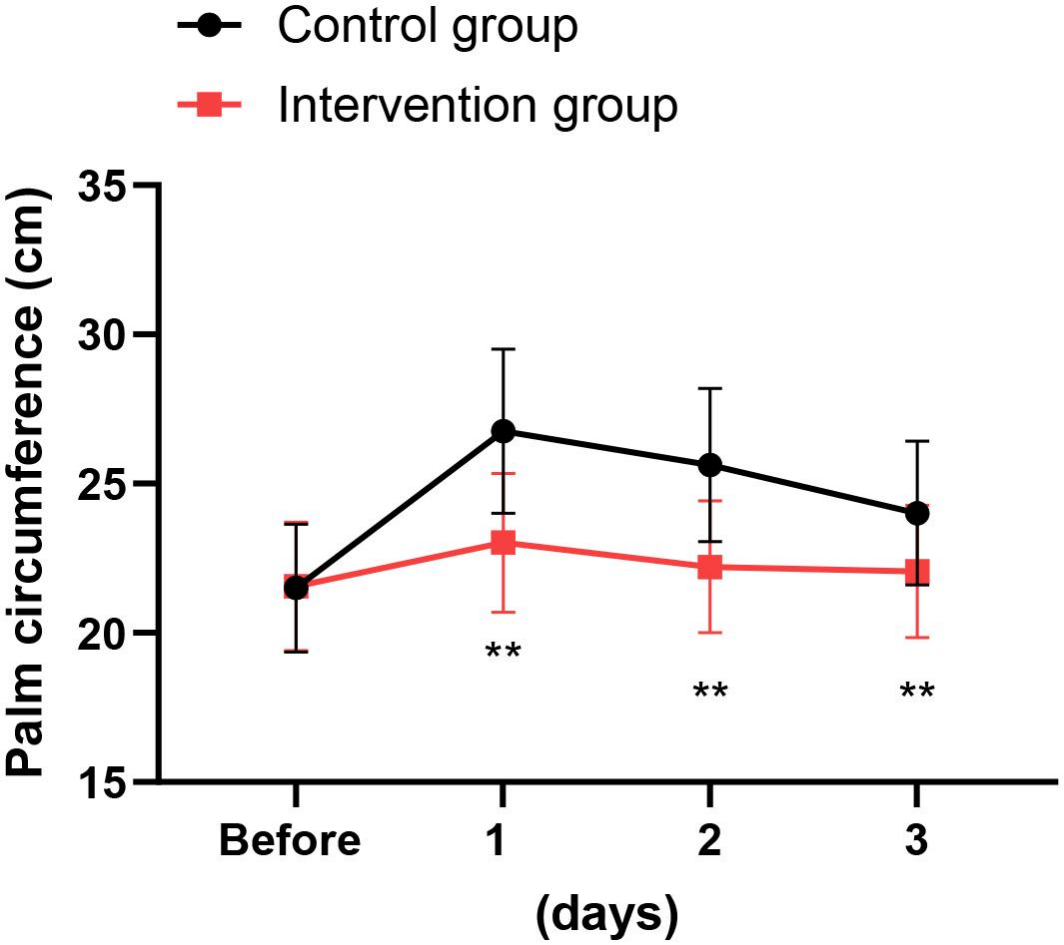
Palm circumference at different times between the two groups. **P < 0.01.

Before the intervention, no significant difference was reported in the palm circumference between the two groups (intervention: 22.5 ± 1.1 cm, control: 22.6 ± 1.0 cm, *P* = 0.64). On the 1st, 2nd, and 3rd days after the intervention, the palm circumference of the intervention group was shorter than that of the control group (all *P* < 0.01, Figure 2). On day 1: intervention 21.8 ± 1.0 cm vs. control 22.3 ± 1.1 cm, mean difference −0.5 cm, 95% CI −0.9 to −0.1, *P* = 0.008; on day 2: intervention 21.2 ± 0.9 cm vs. control 22.0 ± 1.0 cm, mean difference −0.8 cm, 95% CI −1.2 to −0.4, *P* < 0.001; on day 3: intervention 20.8 ± 0.9 cm vs. control 21.9 ± 1.0 cm, mean difference −1.1 cm, 95% CI −1.5 to −0.7, *P* < 0.001.

Within-group analyses revealed significant reductions in palm circumference from baseline to day 3 in both groups (intervention group: mean change −1.7 cm, 95% CI −2.0 to −1.4, *P* < 0.001; control group: mean change −0.7 cm, 95% CI −1.0 to −0.4, *P* < 0.01). The intervention group showed a significantly larger reduction than the control group (between-group difference in change: −1.0 cm, 95% CI −1.4 to−0.6, *P* < 0.01).

### Postoperative bleeding degree within 24 h

No statistically significant difference was reported in the degree of bleeding between the two groups of patients within 24 h after the operation (*P* > 0.05). No bleeding occurred in 44 patients (88%) in the intervention group and 42 patients (84%) in the control group; no obvious bleeding (hematoma ≤2 cm) was observed in 5 patients (10%) vs. 6 patients (12%); obvious bleeding (hematoma >2 cm or requiring re-compression) occurred in 1 patient (2%) vs. 2 patients (4%), respectively (*χ*²=0.71, *P* = 0.70) (Table 3).

**Table 3:** Postoperative bleeding degree within 24 h between the two groups.

Postoperative bleeding degree	Control group (*n* = 50)	Intervention group (*n* = 50)	χ^2^	*P*
No bleeding	44 (88.00)	42 (84.00)	0.71	0.70
No obvious bleeding	5 (22.00)	6 (12.00)		
Obvious bleeding	1 (2.00)	2 (4.00)		

### Incidence of RAO 72 h after surgery

The incidence of RAO at 72 h after surgery was 18.00% (9/50) in the control group and 2.00% (1/50) in the intervention group. The incidence of RAO in the intervention group at 72 h after surgery was lower than that in the control group (*P* = 0.007, Table 4). The absolute risk reduction was 16.0% (95% CI 5.2%–26.8%).

**Table 4:** Incidence of RAO 72 h after surgery between the two groups.

RAO	Control group (*n* = 50)	Intervention group (*n* = 50)	χ^2^	*P*
Yes	9 (18.00)	1 (2.00)	7.111	0.007
No	41 (82.00)	49 (98.00)		

### Comfort level

**Figure 3:**
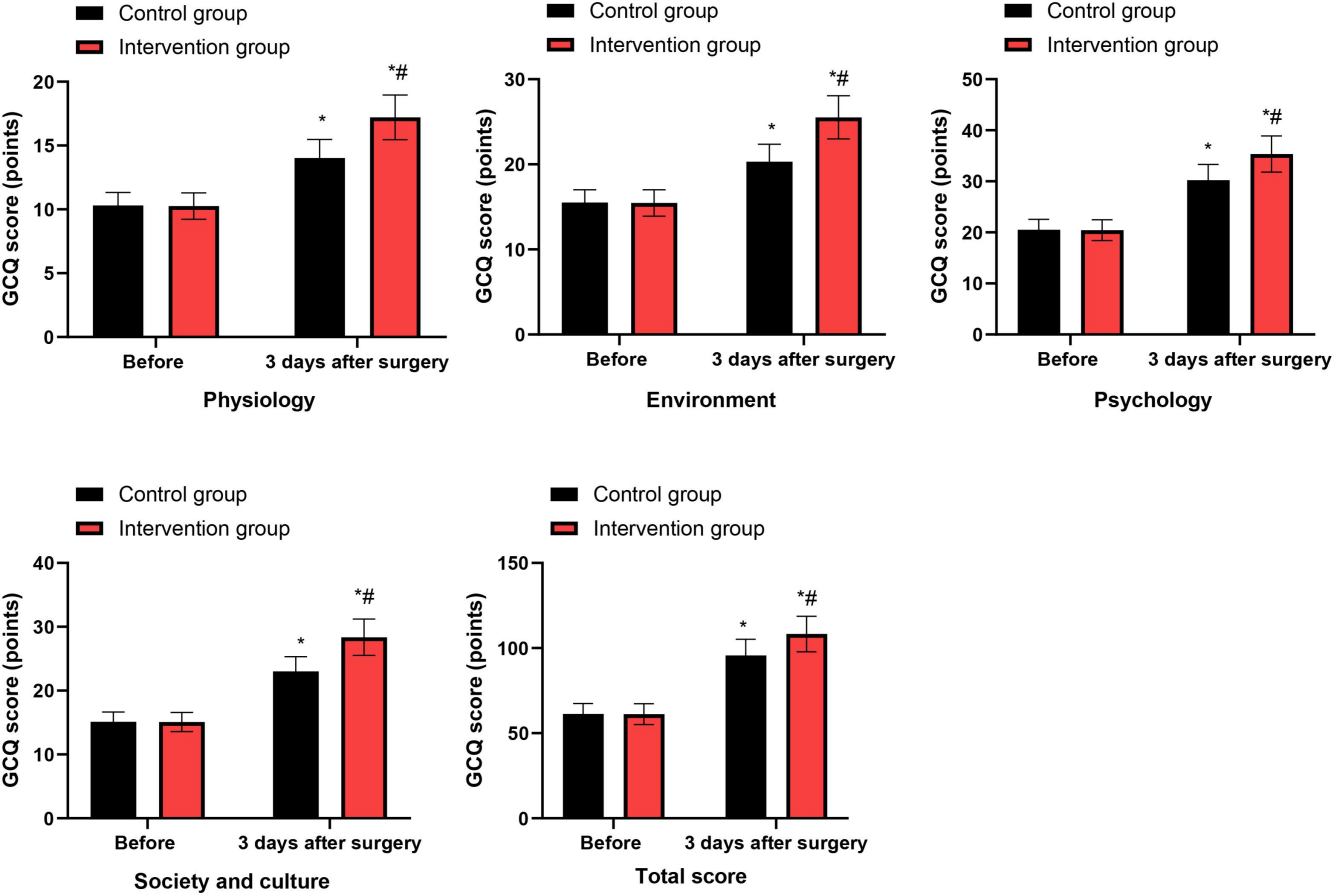
Comfort level between the two groups. *P < 0.05, compared with before intervention; ^#^*P* < 0.05, compared with control group.

Prior to intervention, no significant differences were seen in the GCQ scores between the two groups (**intervention: 68.5** **±** **8.2, control: 67.9** **±** **8.5, *P*** **=** **0.72**). On the 3rd day after the surgery, the GCQ scores of both groups increased compared with their pre-intervention levels (*P* < 0.05). On the 3rd day after the surgery, the GCQ scores of the intervention group were higher than those of the control group (intervention: 95.3 ± 7.6, control: 82.1 ± 8.9, mean difference 13.2, 95% CI 9.8–16.6, *P* < 0.001, Figure 3).

Paired *t*-tests showed significant within-group increases: intervention group mean change +26.8 (95% CI 24.1–29.5, *P* < 0.001); control group mean change +14.2 (95% CI 11.5–16.9, *P* < 0.001). The change-from-baseline score was significantly higher in the intervention group (between-group difference in change: +12.6, 95% CI 9.3–15.9, *P* < 0.001).

### Short-term subjective well-being (assessed by SF-36 at day 3)

**Figure 4:**
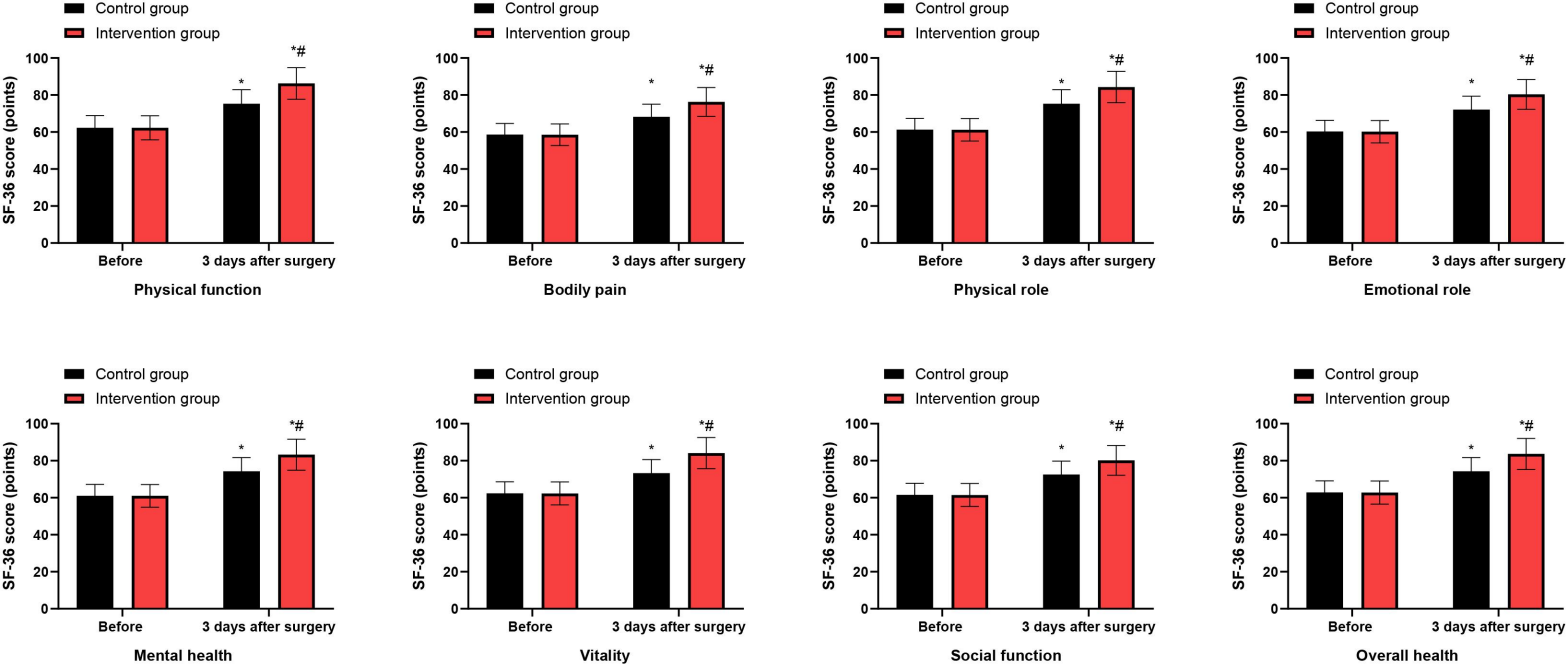
Quality of life between the two groups. *P < 0.05, compared with before intervention; ^#^*P* < 0.05, compared with control group.

Prior to intervention, no significant differences were seen in the SF-36 scores between the two groups (intervention: 62.3 ± 7.5, control: 61.9 ± 7.8, *P* = 0.79). On the 3rd day after the surgery, the SF-36 scores of both groups increased compared to their pre-intervention levels (*P* < 0.05). Furthermore, on the third day after surgery, the intervention group demonstrated higher SF-36 scores than the control group (intervention: 84.6 ± 6.9, control: 73.2 ± 8.1, mean difference 11.4, 95% CI 8.5–14.3, *P* < 0.001, Figure 4).

Paired *t*-tests revealed significant improvements: intervention group mean change +22.3 (95% CI 19.8–24.8, *P* < 0.001); control group mean change +11.3 (95% CI 8.8–13.8, *P* < 0.001). The change—from—baseline score was significantly larger in the intervention group (between-group difference in change: +11.0, 95% CI 7.9–14.1, *P* < 0.001).The mean within-group increase was +22.3 (95% CI 19.8–24.8) in the intervention group and +11.3 (95% CI 8.8–13.8) in the control group, with a between-group difference in change of +11.0 (95% CI 7.9–14.1, *P* < 0.001).

## Discussion

This study explored the impact of a combined nursing intervention utilizing Yunnan Baiyao aerosol and structured finger rehabilitation exercises on local complications following transradial coronary intervention. The findings suggest that, within the short term (3 days), this integrated approach may alleviate postoperative limb pain and swelling, reduce the early incidence of RAO, and improve patient comfort and perceived quality of life, without increasing the risk of postoperative bleeding. However, these findings should be considered preliminary given the short follow-up and single-center design.

The significant reduction in pain scores and palm circumference in the intervention group relative to the control group on postoperative days 1 through 3 suggests a synergistic effect of the applied interventions. Finger rehabilitation exercises, initiated early and performed rhythmically, likely promote venous and lymphatic return, mitigating fluid accumulation in the interstitial spaces—a primary contributor to post-procedural swelling (20). The sequential steps, from massage to active finger movements and acupoint stimulation (Hegu, Houxi), are designed to enhance local circulation and may disrupt the cycle of inflammation and stasis (21). The application of Yunnan Baiyao aerosol complements these mechanical measures. Based on previously documented pharmacological properties (hemostasis, circulation-promotion, anti-inflammation) (12, 22) and clinical reports of its efficacy using the same sequential application method (23), it is hypothesized that the aerosol may act locally to accelerate the absorption of exudate and modulate the inflammatory response. However, because the present study did not measure tissue absorption of the aerosol or any direct pharmacodynamic markers, this mechanism remains speculative and is not validated by our data.

A particularly noteworthy finding is the significantly lower incidence of RAO at 72 h in the intervention group (2.00% vs. 18.00%, *P* = 0.007). RAO is a recognized complication of transradial access, often linked to post-procedural inflammation, thrombosis, and impaired flow (24, 25). The observed reduction may be attributed to the dual action of the intervention. The finger exercises maintain a degree of distal arterial flow and prevent prolonged stasis, while Yunnan Baiyao's anti-thrombotic and circulation-promoting properties may help maintain radial artery patency (26, 27). If confirmed in future studies with longer follow-up, this observation could have clinical implications for preserving radial artery patency for potential future access procedures.

Interestingly, while the intervention reduced swelling and RAO, it did not increase the risk of bleeding, as evidenced by the comparable postoperative bleeding rates between the two groups within 24 h (*P* > 0.05). This indicates that the regimen balances pro-circulatory and anti-thrombotic effects with hemostatic safety, which is crucial in the post-interventional setting.

The improvements in patient-reported outcomes—specifically, higher scores on the GCQ and the SF-36 scale on postoperative day 3 in the intervention group—further support the value of this nursing strategy. Effective alleviation of discomforting symptoms like pain and swelling directly enhances the patient's subjective experience of comfort. Furthermore, the structured nature of the finger exercise regimen, administered by nursing staff, may have provided psychological reassurance and a sense of active participation in recovery, positively influencing overall well-being and satisfaction in the early postoperative period.

The 18% RAO incidence observed in our control group warrants specific discussion. Current European and American consensus guidelines recommend a hemostasis compression duration of approximately 2 h after transradial intervention. A recent 2023 mixed-treatment comparison meta-analysis of 10 randomized trials (4,911 patients) concluded that “a hemostasis duration of 2 h offers the best balance for efficacy (prevention of RAO) and safety (prevention of access site hematoma/rebleeding)” (28). Prolonged compression is a well-established risk factor for RAO; the RESCUE-RAO trial demonstrated that RAO rates were 10.1% with prolonged (8 h) vs. 3.2% with shorter (3 h) hemostasis (*P* < 0.001) (29). Our control group protocol (6 h compression) deviates from current guideline recommendations and likely contributed to the 18% RAO incidence. Consequently, the observed 2.00% vs. 18.00% RAO difference between groups should be interpreted with caution: while it supports the efficacy of the combined intervention, part of the high RAO rate in the control group may be attributable to the prolonged compression protocol rather than solely to the absence of the intervention. Had a 2 h patent hemostasis protocol been used in the control group, the RAO rate would likely have been lower, and the absolute risk reduction attributed to the intervention might have been smaller. This represents a significant limitation of our study.

## Limitations

It is essential to recognize several constraints inherent in this study.

**1. Single**-**center and small sample size.** The study was conducted at a single center with a relatively small sample size (*n* = 100), which limits the generalizability of the findings.
**2. RAO rate in context.** The reported RAO rates typically range from 1% to 10%, a comprehensive review of the literature reveals substantial heterogeneity. A 2024 meta-analysis of 41 studies (30,020 patients) reported an overall RAO incidence of 13% (95% CI: 9%–16%) and an early RAO (within 24 h) incidence of 14% (95% CI: 10%–18%) across 26 studies. Another large-scale systematic review of 66 studies (31,345 participants) found incident RAO ranged from <1% to 33%, varying with the timing of assessment. A SCAI review similarly noted that “the incidence of RAO following coronary angiography has varied from less than 1% to up to 33% in early studies”. Thus, the 18% incidence in our control group falls within the range reported in the literature and is consistent with the upper bound of the 95% confidence interval of the latest meta-analysis estimate. Potential contributing factors include: (1) strict ultrasound screening, which detects asymptomatic occlusions often missed by palpation or plethysmography; (2) the use of occlusive hemostasis (non-patent hemostasis) in the control group, a known predictor of RAO; (3) a standard heparin regimen that may have been suboptimal compared to contemporary high-dose protocols; and (4) assessment at 72 h, which may capture more persistent occlusions than earlier time points. Importantly, the 18% control group incidence and the 2% intervention group incidence yield an absolute risk reduction of 16%, supporting the efficacy of the combined intervention.
**3. Prolonged compression protocol.** The prolonged (6 h) compression protocol used in the control group deviates from current guideline recommendations (target 2 h). This likely elevated the RAO rate in the control group and may have overestimated the absolute treatment effect of the intervention. Future studies should adopt guideline-concordant hemostasis protocols (2 h compression with patent hemostasis) as the control standard to ensure generalizability of findings.
**4. Open**-**label design and lack of blinding.** This was an open-label study. Blinding of patients and nursing staff was not feasible because the Yunnan Baiyao aerosol leaves a visible spray residue and the finger exercises cannot be mimicked by a sham procedure. Consequently, all patient-reported outcomes (pain, comfort, quality of life) are susceptible to bias from expectation or placebo effects, which may have inflated the observed differences. Moreover, outcome assessors (e.g., nurses measuring pain and palm circumference, ultrasonographers evaluating RAO) were also not blinded, introducing detection bias. Although ultrasound provides objective criteria, knowledge of group assignment could have influenced the interpretation of equivocal flow abnormalities; thus, the 2.00% vs. 18.00% RAO incidence should be interpreted with caution.
**5. Combined intervention.** The intervention group received two active components simultaneously (finger exercises and Yunnan Baiyao aerosol), while the control group received neither. This design cannot isolate the individual effect of each component, nor determine whether the observed benefits are synergistic or attributable to a single element. Additionally, the study did not measure tissue absorption, local concentration, or systemic distribution of Yunnan Baiyao aerosol components; therefore, we cannot confirm that the active ingredients reached target tissues or exerted the claimed pharmacological effects. Any reference to these mechanisms in interpreting our findings is based on prior literature and remains hypothetical within the context of this trial.
**6. Short follow**-**up period.** The follow-up period was limited to 3 days. This timeframe is adequate for assessing early complications (acute pain, swelling, very early RAO) but insufficient to evaluate long-term outcomes such as sustained radial artery patency beyond 72 h, late-onset RAO, or delayed complications (e.g., hand weakness, sensory disturbances, late hematoma). The observed 2.00% RAO incidence at 72 h may underestimate the true cumulative incidence over a longer period, as arteries patent at 72 h could subsequently occlude due to intimal hyperplasia or thrombosis. Therefore, our finding of reduced RAO should be interpreted as an early benefit only; the long-term durability remains unknown.
**7. SF**-**36 timing.** The SF-36 was administered only 3 days after the procedure. This instrument is designed to assess health-related quality of life over weeks to months, and its use at a single early time point (day 3) is not standard. Therefore, the observed higher SF-36 scores in the intervention group should be interpreted solely as better early post-procedural subjective well-being, not as evidence of sustained quality of life benefit. Long-term QoL outcomes remain unknown and should be evaluated in future studies with extended follow-up.
**8. Lack of pharmacokinetic data.** The present study did not measure tissue absorption, local concentration, or systemic distribution of Yunnan Baiyao aerosol components. Therefore, we cannot confirm that the active ingredients reached target tissues or exerted the claimed pharmacological effects. Any reference to these mechanisms in interpreting our findings is based on prior literature and remains hypothetical within the context of this trial.
**9. Definition of functional RAO and lack of late follow**-**up scans.** In this study, functional RAO (punctated/speckled flow pattern on color Doppler) was classified as RAO at the 72 h assessment. However, spontaneous recanalization of the radial artery can occur in over 50% of cases with functional RAO, and punctated flow does not necessarily predict permanent occlusion. Without later follow-up scans (e.g., at 30 days), the true rate of persistent RAO may be overestimated, which in turn could overestimate the apparent treatment benefit of the intervention.

## Future research directions

Given these limitations—particularly the open-label design, lack of assessor blinding, combined intervention, and short follow-up—our findings should be considered hypothesis—generating rather than definitive. Future studies should incorporate blinded outcome assessment, patient blinding using a sham aerosol and sham exercises (e.g., gentle hand movements without therapeutic intent), and a factorial (e.g., 2 × 2) or three-arm design to isolate the effects of each component. Longer follow-up (e.g., 30 days, 3 months, or 1 year) is essential to assess durability. Additionally, investigating precise mechanisms—such as changes in inflammatory biomarkers or detailed vascular ultrasound parameters—could provide deeper insight.

## Conclusion

In conclusion, within the limitations of this short-term, open-label, single-center study, the integrated nursing protocol of structured finger rehabilitation exercises combined with Yunnan Baiyao aerosol application was associated with reduced postoperative limb pain and swelling, a lower early incidence of RAO, and improved patient comfort and short-term subjective well-being. This regimen may represent a promising non-invasive adjunct to standard post-procedural care, but these preliminary findings require confirmation in larger, multi-center studies with longer follow-up, blinded assessment, and appropriate timing of QoL measurement.

## Data Availability

The original contributions presented in the study are included in the article/Supplementary Material, further inquiries can be directed to the corresponding author.
